# Systemic immune-inflammation index is associated with high risk for prostate cancer among the U.S. elderly: Evidence from NHANES 2001-2010

**DOI:** 10.3389/fonc.2024.1441271

**Published:** 2024-09-23

**Authors:** Ran He, Youjun Ye, Qilei Zhu, Changsheng Xie

**Affiliations:** ^1^ The First School of Clinical Medicine, Zhejiang Chinese Medical University, Hangzhou, China; ^2^ The Third Clinical Medical College, Zhejiang Chinese Medical University, Hangzhou, China; ^3^ Department of Medical Oncology, The First Affiliated Hospital of Zhejiang Chinese Medical University (Zhejiang Provincial Hospital of Traditional Chinese Medicine), Hangzhou, China

**Keywords:** systemic immune-inflammation index, total prostate-specific antigen, free prostatespecific antigen, fPSA%, prostate cancer, cross-sectional study

## Abstract

**Purpose:**

The Systemic Immuno-Inflammation Index (SII) is a crucial clinical measure of inflammation, and there is currently no solid evidence linking SII to an increased risk of prostate cancer (PCa). Through the analysis of serum total prostate-specific antigen (tPSA), free prostate-specific antigen (fPSA), and the tPSA/fPSA (fPSA%) ratio, this study sought to investigate the relationship between SII and PCa risk among the U.S. elderly.

**Methods:**

Elderly male participants were gathered from the NHANES database between 2001 and 2010.SII was calculated by platelet count * neutrophil count/lymphocyte count. High risk individuals for prostate cancer were defined as those with tPSA > 4 ng/ml and fPSA% < 16%. Multivariate logistic regression models, restricted cubic spline curves, and subgroup analyses were used to assess the relationship between SII and PCa risk.

**Results:**

This research comprised 2664 people in total, 137 (5.14%) of whom were deemed to be at high risk of developing PCa. Multivariate logistic regression analysis, after controlling for variables, revealed a significant positive correlation between high PCa risk and an increase in SII (*p* = 0.009). The RCS suggested a turning point at 9.01. Restricted cubic spline curves revealed a non-linear U-shaped association between SII and high PCa risk (*p* for nonlinear = 0.028). Education level, marital status, PIR, alcohol status, smoking status, rheumatoid arthritis status, and heart problem were not significantly correlated with this positive connection, according to subgroup analyses and interaction tests.

**Conclusion:**

The results of this study suggest that inflammation represented by SII is associated with high PCa risk.

## 1 Introduction

Prostate cancer is the second most common cancer in men worldwide and the fifth leading cause of cancer-related deaths in men, with its incidence and mortality rates rising annually in recent years (1, 2). The measurement of serum prostate-specific antigen (PSA) concentration plays an irreplaceable role in the early screening of prostate cancer. However, various factors, such as smoking habits, can affect PSA levels (3). As a result, widespread PSA screening in men may lead to false positives and the overdiagnosis of non-aggressive conditions (4), and a PSA value between 4 and 10 is a gray area for distinguishing between prostate cancer and benign prostatic diseases. Therefore, current studies often use an fPSA% of less than 0.16 as a standard for high-risk prostate cancer (5, 6). Recent studies have found that novel combinations of serum markers have higher clinical potential for distinguishing between high and low risk of prostate cancer (7).

The Systemic Immune-Inflammation Index (SII) is a unique composite index based on neutrophil, platelet, and lymphocyte counts, which accurately reflects the systemic inflammatory state (8). The interaction between systemic inflammation and local immune response is often considered the seventh hallmark of cancer and has been shown to be involved in the development and progression of various cancers (9). SII objectively reflects the inflammation-immune balance in cancer patients and can thus be considered an effective marker for measuring inflammation levels in cancer patients.

Many studies have shown that inflammation is closely related to the occurrence, development, invasion, and metastasis of prostate cancer (PCa) (10). Research indicates that internal inflammation of the prostate is a risk factor for PCa. The “inflammatory storm” within the prostate has been shown to accelerate the development of PCa by causing DNA damage and overexpression of anti-cancer genes (11, 12). However, there is currently no direct method for assisting prostate cancer screening through inflammation markers.

Recent studies have found a potential positive causal relationship between SII and PCa (13). There is a single-center small-sample retrospective study have further found that SII may be an independent risk factor for diagnosing PCa (14). In addition, research have found a significant association between higher SII levels and poorer prognosis in bladder cancer patients undergoing radical cystectomy (15). Another study based on the UK Biobank found a strong association between SII and the risk of colorectal cancer and lung cancer (16). However, previous studies predicting the relationship between SII and prostate cancer risk based solely on tPSA concentration lack rigor and there is no clear evidence yet on whether SII can serve as a predictive marker for assessing the risk of prostate cancer. Therefore, we collected relevant indicators from the publicly available National Health and Nutrition Examination Survey (NHANES) data from 2001 to 2010. This approach seeks to integrate new inflammatory markers like SII to enhance the precision of prostate cancer screening and risk stratification, offering varied evidence from an inflammatory perspective. It also lays the groundwork for broader studies on the association of SII with the risk of different cancers.

## 2 Materials and methods

### 2.1 Study population

**Figure 1:**
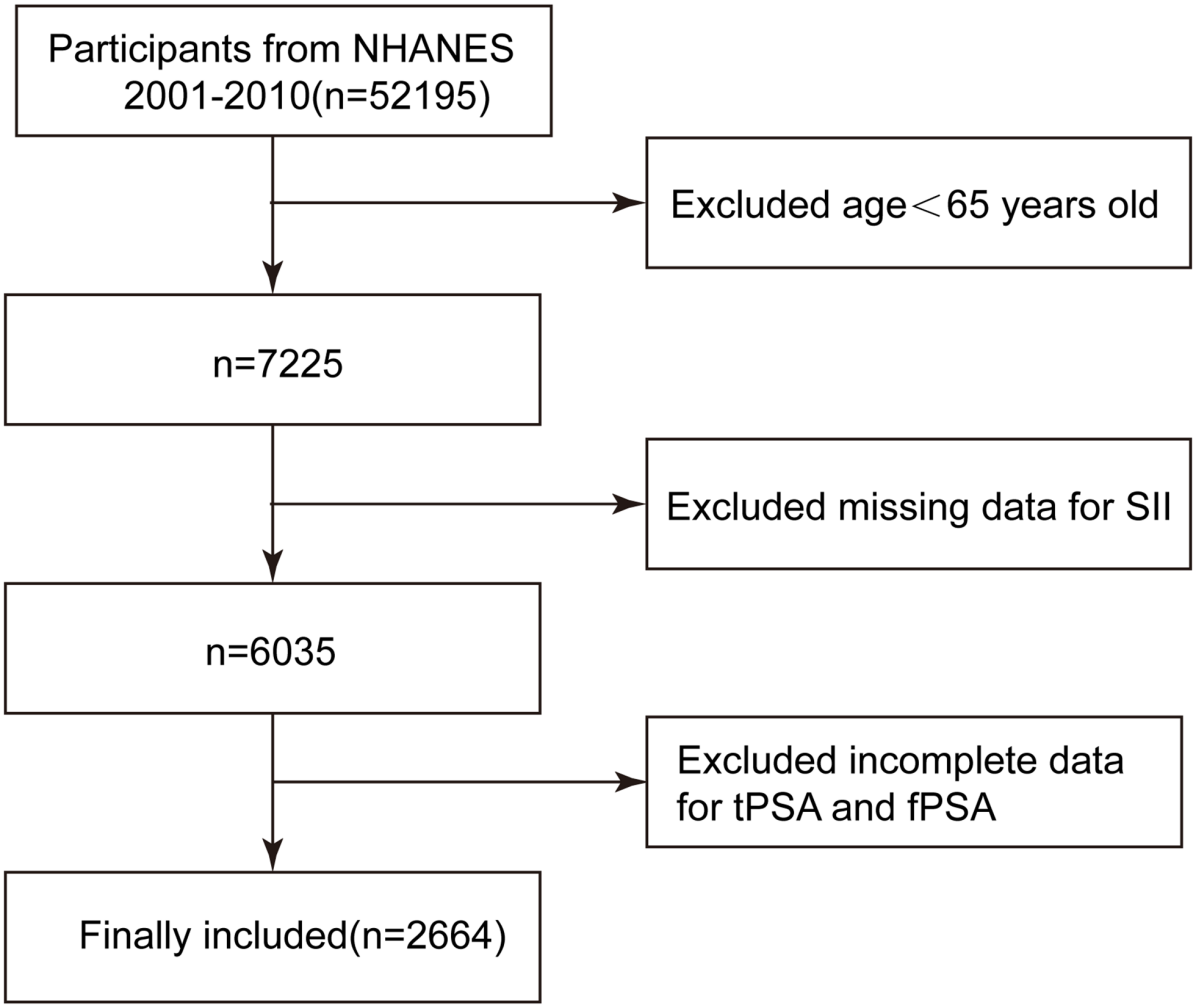
Flowchart of the study participants.

This study is a cross-sectional study based on the population included in the NHANES database from 2001 to 2010. The NHANES database is a project of the National Center for Health Statistics in the United States, dedicated to collecting extensive health information from the population to support various research studies. The NHANES study adheres to the ethical guidelines set forth in the 1975 Helsinki Declaration and received informed consent from all participants. This research was conducted in accordance with the Strengthening the Reporting of Observational Studies in Epidemiology (STROBE) guidelines for cross-sectional studies. A total of 52,195 participants were obtained from the NHANES database from 2001 to 2010, among which 7,225 participants were aged ≥65 years. Among them, 1,190 individuals did not have complete information on platelet (PLT), lymphocyte, and neutrophil counts. After screening, 3,371 participants still lacked complete information on tPSA and fPSA. Therefore, our final analysis included 2,664 participants with complete information. See **Figure 1** for details.

### 2.2 Definitions of SII and the high risk population for prostate cancer

The participants undergoing PSA testing were all 65 years old and above, and individuals with current prostate infection or inflammation, rectal examination within the past week, prostate biopsy within the past month, cystoscopy within the past month, or a history of prostate cancer were excluded. tPSA was obtained using the Hybritech PSA method on Beckman Access, and fPSA was detected using the Access Hybritech free PSA assay kit. Multiple clinical studies have shown that when tPSA is >4 and t/fPSA is less than 16 (17, 18), the probability of patients having prostate cancer greatly increases, and further diagnostic procedures such as enhanced MR or biopsy should be conducted to confirm the diagnosis. This screening method has become the mainstream approach in clinical practice for defining individuals at high risk of prostate cancer. Therefore, we defined such patients as the extremely high risk group for clinical prostate cancer, while others were considered to have a lower risk of prostate cancer.

SII is commonly used to assess patients’ immune function and inflammatory status, with a high SII often indicating immune dysfunction and inflammation progression. According to previous research findings (19, 20), we calculated SII using the formula platelet count * neutrophil count/lymphocyte count. To simplify the calculation of the data, we performed a logarithmic transformation on SII. After consulting earlier research (20)and taking into account the various definitions of SII’s crucial values, we finally split the log-transformed SII into four equal sections using quartile averages: low SII (Q1), low-medium SII (Q2), medium-high SII (Q3), and high SII (Q4).

### 2.3 Covariates

We extensively collected covariates that could potentially influence the relationship between SII and PSA. Individual information included race, education level, marital status, poverty status, BMI, smoking and drinking status. Among them, we distinguished the impoverished population based on whether the PIR was greater than 1. Besides demographic characteristics, laboratory tests known from previous studies to influence prostate cancer—such as cholesterol, total bilirubin, glucose, lactate dehydrogenase, and creatinine—were included to adequately control for confounding variables (21, 22). The specific detection methods for the above laboratory tests are all documented in NHANES. Additionally, extensive research has established that conditions like hypertension, diabetes, stroke, hyperlipidemia, arthritis, and heart disease independently affect the survival of prostate cancer patients (23, 24). Even though some variables did not achieve statistical significance in univariate analysis, they were still included as covariates due to their potential influence, as indicated by earlier studies.

### 2.4 Statistical analysis

Study’s statistical analysis took sample weights into account and used “survey” package to perform weighted computations in R. Continuous variables are described as mean (Standard Deviation), while categorical variables are represented using numbers (Percentages). Differences in all influencing factors between high risk and low risk prostate cancer populations were compared using Kruskal-Wallis rank-sum test for continuous variables and chi-square test for categorical variables. The log-transformed SII was divided into quartiles, with low SII (Q1) as the reference. Multiple linear regression was used to calculate the β values or OR values and corresponding 95% confidence intervals for both unadjusted and adjusted models, thereby examining the significant correlation and trend between different levels of SII and high risk prostate cancer. Restricted cubic spline (RCS) regression models were applied to analyze the 10th, 50th, and 90th percentiles of prostate cancer risk groups, tPSA, fPSA, and fPSA%, to explore linear/non-linear relationships. To further explore potential factors influencing LogSII and high/low risk prostate cancer populations, some covariates that might have an impact were included in subgroup analyses. Regression analyses were performed on adjusted models with categorical variables added as effect modifiers to observe significant interactions between different categories.

In this study, we fitted two adjusted statistical models using regression analysis. In Adjusted I Model, adjustments were made for race, education level, poverty level, marital status, BMI, smoking and drinking status. Adjusted II Model, adjustments were made for the variables included in Adjusted I Model, as well as for cholesterol, total bilirubin, glucose, lactate dehydrogenase, creatinine, hyperlipidemia status, diabetes status, stroke status, rheumatoid arthritis status, and heart disease status. Adjusted I Model focuses on adjusting for demographic and lifestyle factors to minimize their confounding effects on the outcomes. Building on this, Adjusted II Model includes additional adjustments for laboratory tests and comorbidities, representing the fully adjusted model in this study. This comprehensive adjustment allows for a more accurate estimation of odds ratio (OR), enhancing the robustness of the findings. All statistical analyses were performed using R version 4.2.3. A *p* value less than 0.05 was considered statistically significant.

## 3 Results

### 3.1 Baseline characteristics of the study population

From 2001 to 2010, a total of 2,664 elderly participants were included in our study, with an average age of 73.99 years. Among them, the population with a high incidence rate of prostate cancer accounted for 5.14% of the total population. The average levels of tPSA and fPSA in the included population were 3.00 and 0.70, respectively, while the fPSA% was 29.99%. The average value of LogSII in the included population was 9.02. In the baseline characteristic table stratified by high and low risk of prostate cancer, we found no significant statistical differences in Educational attainment, Poverty status, Drink status, Smoking status, total Bilirubin, Glucose, Lactate dehydrogenase level, Creatinine, Hyperlipidemia status, Diabetes status, Arthritis or rheumatism status, and Stroke between groups, while significant differences were observed in the remaining characteristics. See **Table 1** for details.

**Table 1:** Baseline characteristics of participants in NHANES 2001-2010 (weighted).

Characteristic	Group		*P* value
	Low PCa risk(n=2527)	High PCa risk(n=137)	
Age,years	73.92 ± 5.97	75.45 ± 6.29	0.0208
Race and ethnicity,%			0.004
Mexican American	350 (13.85%)	15 (10.95%)	
Other Hispanic	110 (4.35%)	9 (6.57%)	
Non-Hispanic White	1666 (65.93%)	74 (54.01%)	
Non-Hispanic Black	332 (13.14%)	35 (25.55%)	
Other Race	69 (2.73%)	4 (2.92%)	
Educational attainment,%			0.2156
Less Than 9th Grade	589 (23.31%)	38 (27.74%)	
9-11th Grade	364 (14.40%)	23 (16.79%)	
High School Grad/GED or Equivalent	566 (22.40%)	25 (18.25%)	
Some College or AA degree	500 (19.79%)	25 (18.25%)	
College Graduate or above	508 (20.10%)	26 (18.98%)	
Weight status(BMI),kg/m2	28.16 ± 4.87	26.16 ± 4.71	<0.001
Poverty(PIR),%			0.3413
≤1	337 (13.34%)	24 (17.52%)	
>1	2190 (86.66%)	113 (82.48%)	
Marital status,%			0.0027
Married	1779 (70.40%)	80 (58.39%)	
Living Without partner	707 (27.98%)	55 (40.51%)	
Living with partner	41 (1.62%)	2 (1.46%)	
Drink status,%			0.9089
Former&Current	535 (21.17%)	35 (25.55%)	
Never	1992 (78.83%)	102 (74.45%)	
Smoking status,%			0.1553
Former&Current	304 (12.03%)	26 (18.98%)	
Never	2223 (87.97%)	111 (81.02%)	
Cholesterol,mmol/L	4.81 ± 1.04	5.01 ± 1.03	0.0113
Bilirubin(total),umol/L	14.49 ± 5.56	14.38 ± 5.42	0.5917
Glucose,mmol/L	6.11 ± 2.21	6.29 ± 2.37	0.7122
Lactate dehydrogenase,U/L	137.43 ± 31.24	140.08 ± 35.30	0.6512
Creatinine,umol/L	103.28 ± 51.86	104.44 ± 50.96	0.3222
Hyperlipidemia,%			0.5341
Yes	1436 (56.83%)	69 (50.36%)	
No	1091 (43.17%)	68 (49.64%)	
Diabetes,%			0.415
Yes	532 (21.05%)	12 (16.06%)	
No	1928 (76.30%)	112 (81.75%)	
Borderline	67 (2.65%)	3 (2.19%)	
Stroke,%			0.4557
Yes	266 (10.53%)	12 (8.76%)	
No	2261 (89.47%)	125 (91.24%)	
Arthritis or rheumatism,%			0.9113
Yes	429 (16.98%)	19 (13.87%)	
No	2098 (83.02%)	118 (86.13%)	
Heart problem,%			0.0283
Yes	1072 (42.42%)	43 (31.39%)	
No	1455 (57.58%)	94 (68.61%)	
tPSA,ng/mL	2.37 ± 3.11	14.77 ± 20.87	<0.001
fPSA,ng/mL	0.65 ± 0.83	1.59 ± 2.44	<0.001
fPSA%,%	31.00 ± 12.13	11.37 ± 3.06	<0.001
LogSII	9.01 ± 0.87	9.21 ± 1.08	0.0018

### 3.2 SII is associated with increased likelihood of the risk for PCa

Through multivariable logistic regression analysis, we found that higher levels of SII were associated with the high risk group of prostate cancer (**Table 2**). This association was significant in the Non-adjusted model, Adjust I model, and Adjust II model. In the Adjust II model, the likelihood of high risk prostate cancer was significantly increased by 96% for participants in the highest LogSII quartile compared to those in the lowest LogSII quartile. Moreover, as LogSII increased, the likelihood of being at high risk for prostate cancer also showed an upward trend (*p* for trend = 0.009).

**Table 2:** Associations of LogSII with PCa risk and serum PSA levels by linear regression in NHANES 2001-2010 (weighted).

	Quartiles of LogSII levels				
	3.57 - 8.519	8.519 - 9.0255	9.0255 - 9.5332	9.5332 - 13.514	*P* for trend
Risk group					
Non-adjusted	1	0.65 (0.32, 1.30) 0.220	0.78 (0.45, 1.35) 0.365	1.78 (1.15, 2.76) 0.010	0.008
Adjust I^a^	1	0.78 (0.38, 1.57) 0.472	0.87 (0.49, 1.52) 0.615	1.89 (1.18, 3.01) 0.009	0.008
Adjust II^b^	1	0.80 (0.39, 1.65) 0.531	0.88 (0.49, 1.57) 0.662	1.96 (1.19, 3.24) 0.009	0.009
tPSA					
Non-adjusted	0	0.30 (-0.09, 0.70) 0.133	0.56 (0.07, 1.05) 0.025	0.98 (0.57, 1.39) <0.001	<0.001
Adjust I	0	0.47 (0.06, 0.88) 0.026	0.71 (0.18, 1.25) 0.010	1.05 (0.62, 1.48) <0.001	<0.001
Adjust II	0	0.54 (0.13, 0.95) 0.010	0.78 (0.23, 1.33) 0.006	1.09 (0.66, 1.52) <0.001	<0.001
fPSA					
Non-adjusted	0	0.06 (-0.03, 0.14) 0.192	0.10 (0.01, 0.18) 0.021	0.12 (0.04, 0.20) 0.003	0.002
Adjust I	0	0.08 (-0.01, 0.17) 0.094	0.11 (0.03, 0.20) 0.009	0.13 (0.04, 0.21) 0.003	0.003
Adjust II	0	0.09 (-0.00, 0.18) 0.059	0.12 (0.04, 0.21) 0.006	0.13 (0.05, 0.21) 0.003	0.002
fPSA%					
Non-adjusted	0	-1.42 (-2.23, -0.62) 0.036	-2.21 (-3.02, -1.41) 0.014	-2.94 (-3.75, -2.14) <0.001	<0.001
Adjust I	0	-2.19 (-3.66, -0.72) 0.004	-2.30 (-3.77, -0.83) 0.003	-4.02 (-5.58, -2.46) <0.001	<0.001
Adjust II	0	-2.16 (-3.66, -0.67) 0.006	-2.32 (-3.78, -0.86) 0.002	-4.17 (-5.74, -2.59) <0.001	<0.001

^a^Adjust I: Adjusted for age, race, education level, poverty level, marital status, BMI, smoking and drinking status.

^b^Adjust II: model 1+ cholesterol, total bilirubin, glucose, lactate dehydrogenase, creatinine, Hyperlipidemia status, diabetes status, stroke status, rheumatoid arthritis status, and heart disease status.

Additionally, we conducted multivariable linear regression analyses on LogSII quartiles and tPSA, fPSA, and fPSA%. Higher LogSII levels were associated with higher levels of tPSA and fPSA, and this association was significant in Adjust II model. In the Adjust II model, tPSA increased by 1.09 and fPSA increased by 0.13 for participants in the highest LogSII quartile compared to those in the lowest LogSII quartile. Moreover, there was an upward trend observed in the tPSA levels as LogSII increased (*p* for trend < 0.001). There was also an increased trend in the fPSA (*p* for trend = 0.003).

Higher LogSII quartiles were associated with lower levels of fPSA%, and this association was significant in all three models for participants in the second, third, and highest quartiles compared to those in the lowest LogSII quartile. In the Adjust II model, fPSA% decreased by 4.17% for participants in the highest LogSII quartile compared to those in the lowest LogSII quartile. Additionally, as LogSII increased, the levels of fPSA% showed a downward trend (*p* for trend < 0.001).

### 3.3 The nonlinear relationship between SII and the risk for PCa

**Figure 2:**
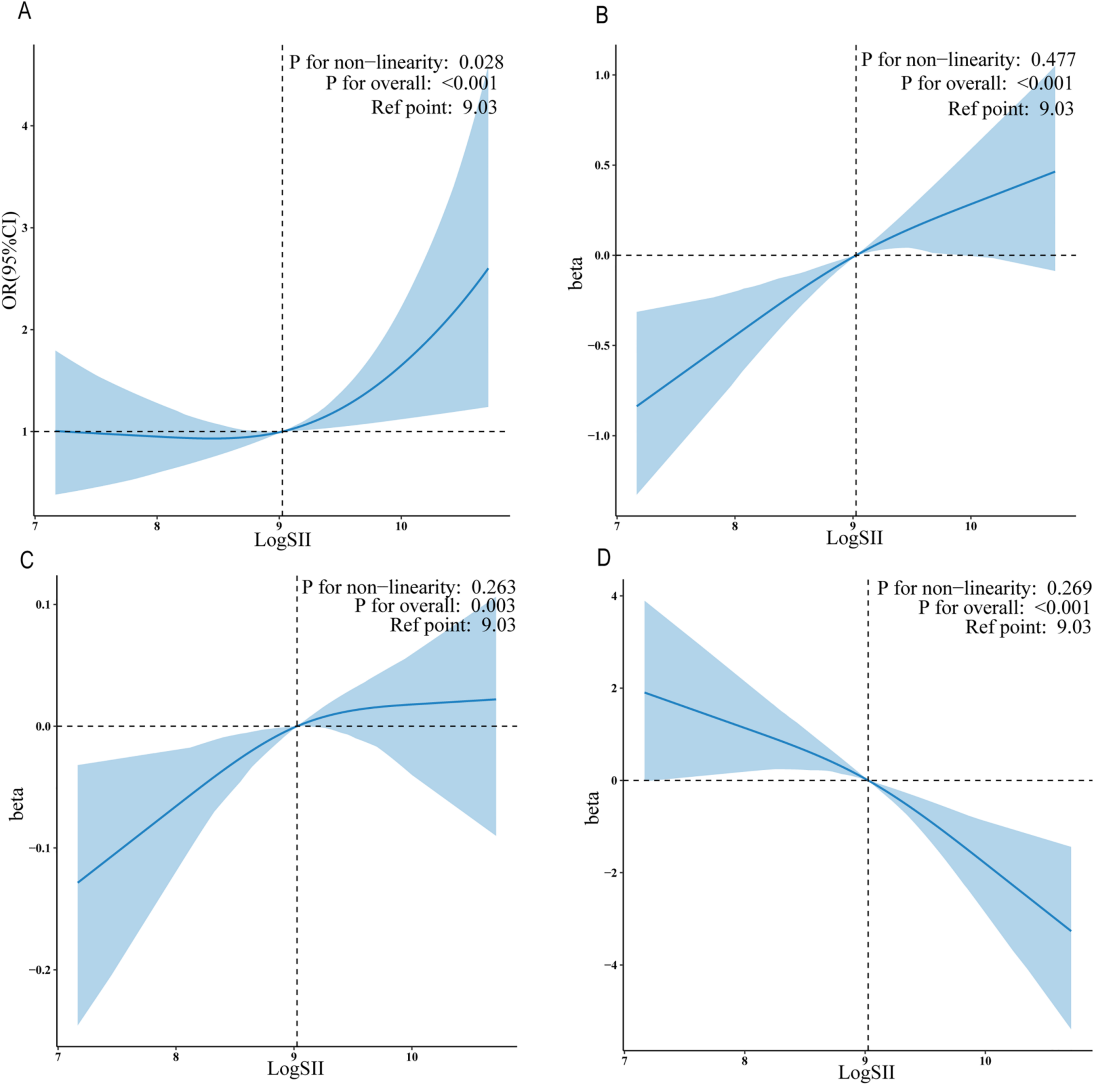
The exposure-response associations of the SII with serum PSA levels and PCa by restricted cubic spline model (weighted). Association of LogSII with the PCa risk group **(A)**, tPSA **(B)**, fPSA **(C)** and fPSA% **(D)**.

For the Adjust II model, we used regression cubic splines to demonstrate the relationship between LogSII and PCa risk groups (**Figure 2A**), tPSA (**Figure 2B**), fPSA (**Figure 2C**), and fPSA% (**Figure 2D**). LogSII showed a linear positive correlation with tPSA and fPSA, and a linear negative correlation with fPSA%. There was a non-linear positive correlation between LogSII and high/low PCa risk groups (*p* for nonlinear = 0.030).

Furthermore, we identified that a LogSII value of 9.03 may serve as a clinically significant threshold. Results from the aforementioned RCS analysis consistently indicated that 9.03 is the inflection point of the threshold. When LogSII is less than 9.03, there is no significant association with a high risk of prostate cancer. However, when LogSII exceeds 9.03, a significant positive correlation with a high risk of prostate cancer is observed. This overall relationship exhibits a non-linear U-shaped pattern.

Association of LogSII with the PCa risk group (A), tPSA (B),fPSA (C)and fPSA%(D).

### 3.4 Subgroup analysis

**Figure 3:**
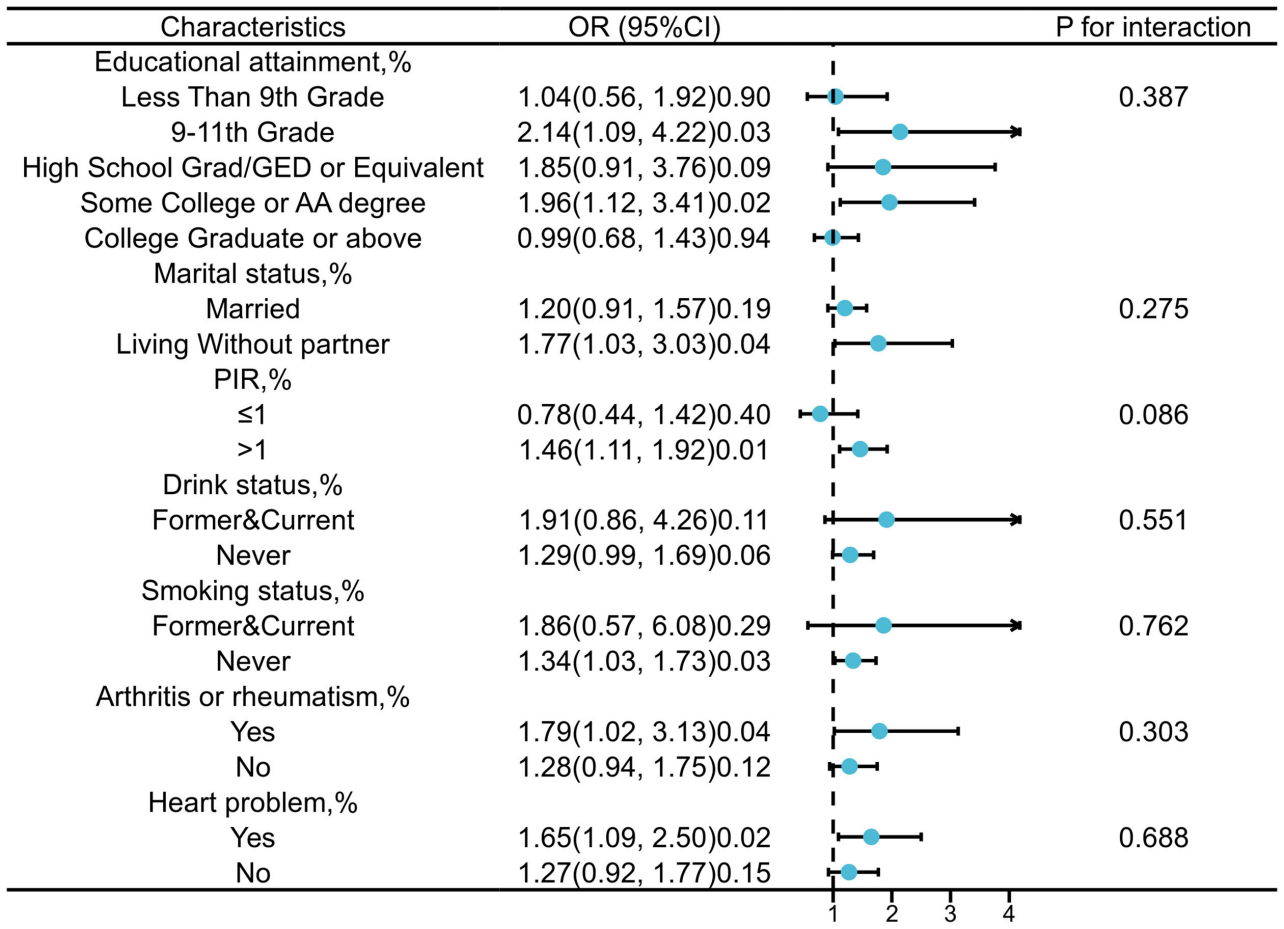
Forest plot for subgroup analysis of association between SII and PCa risk (weighted).

The stratified analysis based on education level, marital status, PIR, alcohol status, smoking status, rheumatoid arthritis status, and heart problem was conducted to further investigate the relationship between SII and PCa (**Figure 3**). Interaction tests indicated that there were no statistically significant differences in the association between SII and PCa risk across the different strata, suggesting that these stratifying factors do not have a statistically significant impact on the observed positive correlation. Nonetheless, we observed higher OR values in subgroups with PIR greater than 1, a history of smoking, alcohol consumption, rheumatoid arthritis, and heart problem.

## 4 Discussion

This study collected 2664 participants aged 65 and above from the NHANES database to explore the association between SII and PCa risk in high and low risk groups. The findings indicated a positive linear correlation between SII and both tPSA and fPSA. Additionally, a notable negative linear correlation was observed between SII and the percentage of fPSA%. However, it exhibited a non-linear U-shaped association with high and low risk PCa subgroups. Subgroup analysis revealed that variables like race, marital status, and stroke status, among others, did not significantly differ between subgroups.

Previous studies utilizing NHANES data have investigated the relationship between the Systemic Immune-Inflammation Index (SII) and prostate cancer, using the incidence of prostate cancer as the outcome measure. Although the study found that SII was associated with a 7% increased risk of prostate cancer, this association did not reach statistical significance (25). Some reports have identified SII as a significant diagnostic marker for patients with PSA levels below 10 ng/ml in prostate fusion biopsy (26), with the combined diagnostic efficiency of SII and PSA for prostate cancer surpassing that of PSA alone (27). Conversely, Murray et al. argued that SII cannot distinguish clinically significant prostate cancer from indolent cancer or benign diseases during the initial biopsy (28). As a result, the clinical findings on the relationship between SII and prostate cancer or PSA remain controversial. This study observed that a significant increase in SII levels is associated with a higher risk of prostate cancer. SII could serve as a crucial marker for distinguishing between high-risk and low-risk prostate cancer, offering potential value in supporting prostate cancer diagnosis.

Inflammation has long been recognized as a crucial factor influencing the development and progression of prostate cancer (PCa). Numerous clinical studies have demonstrated the association between inflammation and the development and progression of PCa (29). A retrospective study from Korea, which analyzed 746,176 patients, found a significant association between prostatic inflammation and an increased incidence of PCa, with acute prostatitis posing a higher risk than chronic inflammation (30). Furthermore, a study based on U.S. Medicare data from 1999 to 2010, involving 2,701,782 osteoarthritis patients, 13,044 ankylosing spondylitis patients, and 10,859,304 controls, found that both osteoarthritis and ankylosing spondylitis increase the risk of developing prostate cancer (31).

The mechanisms by which inflammation induces the occurrence of prostate cancer (PCa) remain a hot research topic. One mainstream view of the inflammation-cancer transformation mechanism is that long-term chronic inflammation promotes mutations in human cells and the genome (32). Reports indicate that the overexpression of inflammation-related genes, along with prolonged activation of various inflammatory signaling pathways, growth proteins, and cellular messengers, promotes cellular mutations and structural variations, leading to prostate cancer as well as castration resistance, metabolic reprogramming, and immunosuppression (33). Furthermore, research indicates that chronic inflammation can influence the tumor immune microenvironment and the urinary microbiome (34, 35). Inflammatory reactive oxygen species (ROS) can mediate oxidative stress, leading to prostatic inflammatory atrophy, which eventually contributes to the development of prostatic adenocarcinoma (36). SII is derived from the counts of neutrophils, platelets, and lymphocytes. Studies have identified neutrophil polarization as a critical factor in cancer development (37), with neutrophils not only producing ROS that cause DNA mutations but also generating inflammatory cytokines that create an immunosuppressive environment (38). Platelets and their byproducts can impact the coagulation cascade, activate oncogenic mutations, and maintain proliferative signals, while also inducing angiogenesis via factors like vascular endothelial growth factor, which promotes tumor metastasis (39). Lymphocytes are intricately linked to cancer; their reduction can lead to immune evasion and tumor progression, whereas their abnormal proliferation may exacerbate immune-related adverse events (40).

Compared with previous studies, this study revealed new associations between SII and high and low risk prostate cancer populations, as well as the association patterns with different prostate cancer biomarkers, providing a deeper understanding of the relationship between immunity, inflammation, and prostate cancer risk, and bringing a new perspective to research on prostate cancer screening. This study quantitatively analyzed the potential relationship between SII and prostate cancer risk by calculating the significant inflection point at which SII is associated with prostate cancer risk. This finding provides a new perspective on the contributing factors to prostate cancer risk and may pave the way for more refined screening and diagnostic strategies in the future. Additionally, the large sample size of this study enhances the reliability of its conclusions. Subgroup analyses also highlighted the influence of age and marital status. However, before SII can be adopted as a routine clinical marker, further longitudinal studies and validations across different populations are essential. Longitudinal cohort studies can monitor how SII levels change over time and their impact on prostate cancer risk, while randomized controlled trials can assess the effectiveness of interventions targeting SII in reducing this risk. These approaches will not only strengthen the evidence for a causal relationship but also explore whether SII could be a viable target for preventive strategies. This will be crucial in confirming the reliability of SII in predicting prostate cancer risk and ensuring its practical application.

This study has several limitations. The cross-sectional design of the study limits the ability to draw causal conclusions. While analysis reveals associations between the SII and PCa risk, it cannot infer causality due to the simultaneous measurement of exposure and outcome variables. The reliance on data from the 2001-2010 NHANES survey may not fully reflect recent trends in PSA screening practices or the latest advancements in prostate cancer management. Although these historical data provide valuable insights into the relationship between SII and PCa risk, the temporal gap could affect the generalizability of our findings to the current population. Additionally, due to the lack of data on prostate cancer subtypes and stage, we were unable to analyze the specific impact of pathological types and tumor stages. Furthermore, as this is a cross-sectional study, it precludes further discussion incorporating patients’ subsequent diagnostic outcomes. The exclusion criteria were established to ensure the reliability and accuracy of our analyses by focusing on participants with complete and valid data. However, excluding individuals with incomplete data could introduce selection bias, as it may result in a study sample that is not fully representative of the general population. In cross-sectional studies, data are gathered at one specific time point, a method frequently used in epidemiological research with the NHANES database. However, since factors like platelet, lymphocyte, and neutrophil counts, along with tPSA and fPSA levels, can vary over time, this might introduce some variability in the results. Finally, although we controlled for some confounding variables, we could not account for other potential unknown confounders in this study.

## 5 Conclusion

The results of this study indicate that among elderly, SII is significantly positively correlated with serum levels of tPSA and fPSA, and significantly negatively correlated with fPSA%. It also shows a nonlinear U-shaped correlation with high risk PCa. SII may serve as an effective indicator for identifying high risk populations for PCa from an inflammatory perspective. Further prospective studies are needed to confirm our conclusions.

## Data Availability

In this study, publicly accessible datasets were evaluated. Visit https://www.cdc.gov/nchs/nhanes/index.htm to get this data.
